# Hypoxia Promotes the In Vitro Proliferation of Buffalo Spermatogonial Cells by Increasing Lactate and H3K18la Lactylation Levels

**DOI:** 10.3390/cells14110832

**Published:** 2025-06-03

**Authors:** Mengqi Li, Yanyu Ma, Shenzhi Wang, Haiying Zheng, Chunyan Yang, Anqin Duan, Benliang Zhou, Jianghua Shang, Xingwei Liang, Xiaogan Yang

**Affiliations:** 1 College of Animal Science and Technology, Guangxi University, Nanning 530004, China; pokerqi1992@gmail.com (M.L.); yyma1009@163.com (Y.M.); wangshenzhi9927@163.com (S.W.); duanaq321@163.com (A.D.); zhoubliang@outlook.com (B.Z.); xwliang@gxu.edu.cn (X.L.); 2 Guangxi Key Laboratory of Buffalo Genetics, Reproduction and Breeding, Guangxi Buffalo Research Institute, Chinese Academy of Agricultural Sciences, Nanning 530001, China; haiyingzheng@126.com (H.Z.); ycy3106@163.com (C.Y.); 3 Key Laboratory of Buffalo Genetics, Breeding and Reproduction Technology, Ministry of Agriculture and Rural Affairs, Nanning 530001, China; 4 Guangxi Key Laboratory of Animal Breeding, Disease Control and Prevention, Nanning 530004, China

**Keywords:** spermatogonial cells, hypoxia, proliferation, histone lactylation, buffalo

## Abstract

Hypoxia benefits the proliferation and maintenance of animal spermatogonial cells; however, the underlying mechanism remains incompletely understood. This study aims to investigate the role and mechanism of the hypoxia–glycolysis–histone lactylation axis in the proliferation of buffalo spermatogonial cells (bSCs). bSCs were cultured under different oxygen concentrations to observe changes in cell proliferation. RNA-seq was used to analyze gene expression and signaling pathways. Changes in lactylation were monitored, and CUT&Tag-seq was utilized to determine the regulatory effects of lactylation on gene expression. The glycolytic pathway was regulated to validate the results of the bioinformatic analysis. Oxygen concentrations between 2.5% and 10% support the proliferation of bSCs, with 5% having the most pronounced effect. An amount of 5% oxygen significantly increased the proliferation and pluripotency of bSCs while also promoting glycolysis and lactylation. Inhibition of glycolysis eliminated the proliferative effects of hypoxia. By analyzing genes associated with the key lactylation site H3K18la using CUT&Tag technology, we found that it is closely linked to genes involved in the regulation of proliferation. After inhibition of HK-2 expression, cell proliferation, H3K18la expression, and the expression of these target genes were all suppressed. Hypoxia promotes the proliferation of bSCs via activation of glycolysis, leading to an increase in H3K18la and altered expression of its target genes.

## 1. Introduction

Spermatogonial stem cells (SSCs) are ideal seeded cells [1] for developing livestock breeds and preserving genetic resources, as they play a crucial role in male reproduction and can be manipulated for genetic improvement [2,3]. Effective in vitro culture of SSCs is critical for their industrial application in livestock [4,5]. To date, the in vitro culture system for buffalo SSCs is insufficient, hindering their application in breeding practices [6,7]. This limitation stems from our incomplete understanding of the mechanisms that govern self-renewal and the maintenance of stemness in SSCs [8,9]. An effective in vitro culture system for buffalo spermatogonial cells (bSCs) has not yet been established, primarily due to the challenges in accurately determining their metabolic requirements [10,11].

Hypoxia is an important characteristic of the adult stem cell (ASC) niche [11] and plays a crucial role in regulating various functions. Hypoxia-inducible factors (HIFs), particularly HIF-1α, serve as central regulators that enable cells to sense and respond to low oxygen by activating genes involved in angiogenesis, metabolism, proliferation, and survival [12]. Stem cells such as hematopoietic stem cells (HSCs) in the bone marrow reside in hypoxic niches that help maintain their stemness and regulate their proliferation by promoting glycolytic metabolism over oxidative phosphorylation, thus protecting them from oxidative stress and DNA damage [13,14]. Induced pluripotent stem cells (iPSCs) cultured under hypoxic conditions showed a positive correlation between hypoxia and cellular quality [15]. Other examples of cells requiring hypoxia include keratinocytes, lymphocytes, mesenchymal stem cells, and various progenitor cells involved in tissue regeneration and repair. These cells leverage hypoxic conditions to maintain pluripotency, control differentiation, and support tissue homeostasis. Furthermore, many differentiated cells do not require hypoxia and may even experience reduced proliferation or impaired function under low oxygen conditions. For instance, mature cardiomyocytes require high oxygen levels to maintain contractile function, and hypoxia can lead to impaired contractility and cell damage [16]. Similarly, neurons are highly sensitive to oxygen deprivation, as their high energy demands are primarily met through aerobic metabolism [17]. The testes exhibit low oxygen levels to support SSCs by promoting glycolysis and protecting against oxidative stress, which is essential for spermatogenesis [18,19,20]. Similar hypoxic niches exist in organs like bone marrow and the brain, where low oxygen preserves stemness and regulates proliferation [21,22,23]. This shared hypoxia reflects an evolutionary adaptation to balance oxygen supply and cellular needs, optimizing tissue maintenance and function. Recent studies have indicated that the oxygen concentration is a critical factor that affects SSC behavior, particularly under hypoxic conditions [24]. Similar to culture in 21% oxygen, maintaining SSCs in environments with 3.5% oxygen has been shown to support colony formation, whereas severe hypoxia (1% and 0.1% oxygen) strongly impairs the ability of these cells to proliferate and form germ cell clusters [18]. Hypoxia is crucial for maintaining stem cells by activating glycolysis through hypoxia-sensing molecules [25,26], suggesting a strong connection between glycolysis and the proliferation and pluripotency of stem cells. Overall, hypoxia acts as a critical environmental cue that shapes cellular behavior, particularly in cells with high proliferative or regenerative capacity, by orchestrating adaptive responses that optimize survival and function under low oxygen availability.

Hypoxia regulates pluripotency by activating HIFs that promote glycolytic metabolism, enhance expression of key stemness genes [27], and induce epigenetic changes to maintain stem cell self-renewal and inhibit differentiation [28]. Developmentally and evolutionarily, hypoxic microenvironments form within organs to protect stem cells from oxidative damage, support tissue renewal, and enable controlled differentiation, thereby facilitating proper organogenesis and adaptation to fluctuating oxygen levels [29,30]. Under hypoxic conditions, cells shift their metabolism toward glycolysis to generate ATP [26], leading to elevated lactate levels that contribute to histone lactylation, which strongly affects gene expression and cellular proliferation [31,32]. In iPSCs, the transcription factor GLIS1 induces pluripotency through a signaling cascade involving epigenomic and metabolic remodeling, facilitating the reprogramming of senescent cells into pluripotent cells while promoting genome stability by increasing glycolysis and promoting H3K18la lactylation [33]. Dux increases the reprogramming efficacy of iPSCs by inducing H3K18 lactylation, which regulates the metabolism-H3K18la-mesenchymal-epithelial transition network [34]. The discovery of histone lactylation suggests a regulatory mechanism linking lactate production and metabolism to stem cell pluripotency [35,36]. However, whether histone lactylation can regulate spermatogonial cells proliferation and the maintenance of stemness in livestock, as well as the underlying regulatory mechanisms involved, is unclear.

This study explored the optimization of an in vitro culture system for bSCs by reducing the oxygen concentration to investigate whether the activation of glycolysis induced by hypoxia resulted in lactate accumulation, which subsequently caused changes in histone lactylation. Furthermore, we conducted a combined CUT&Tag-seq and RNA-Seq analysis to further investigate the target genes and mechanisms by which histone lactylation affects bSCs proliferation.

## 2. Materials and Methods

### 2.1. Buffalo Testicle Collection

For this study, we collected testes from three 2-year-old water buffaloes and five 5-month-old water buffaloes. These specimens were provided by the Guangxi Buffalo Research Institute. Prior to the collection of the testes, the buffaloes were anesthetized to ensure humane handling. All procedures related to the collection of animal materials were reviewed and approved by the Animal Experiment Ethics Review Committee of Guangxi University.

### 2.2. Culture of bSCs at Multiple Oxygen Concentrations

The isolation of bSCs was conducted following our previously published protocols [8], utilizing a two-step digestion method involving type IV collagenase and trypsin to digest 5-month-old buffalo testicular tissue into a primary single-cell suspension. After digestion, bSCs were purified via the gelatin differential adhesion method. During this process, cells that remained in suspension after 12 h of in vitro culture were considered bSCs, while those that had adhered were identified as Sertoli cells. Consistent with our previously published method [8], the Sertoli cells, after treatment with mitomycin, could be used as feeder layer cells. The bSCs were subsequently cocultured with mitomycin- treated Sertoli cells in a noncontact manner via a cell culture insert system (Biofil, Guangzhou, China, TCS016012). The basal culture medium used was DMEM/F12 (Gibco, Carlsbad, CA, USA, 11320033), supplemented with fetal bovine serum (FBS) (Gibco, A5670701) and various growth factors, including glial cell-derived neurotrophic factor (GDNF) (R&D Systems, Minneapolis, Minnesota, USA, 212-GD), GDNF family receptor alpha-1 (GFRα1) (R&D, 560-GR), fibroblast growth factor 2 (FGF2) (R&D, 233-FB), and leukemia inhibitory factor (LIF) (R&D, 7734-LF), to promote cell growth and maintenance in vitro. This approach facilitated the long-term culture of bSCs while ensuring their viability and functionality. The gases used for cell culture at different oxygen concentrations were prepared by Guangdong Huate Gas Co., Ltd. (Foshan, China), in accordance with the GB/T 5274.1-2018 standard [37]. The company produced mixed gases with oxygen concentrations of 1%, 2.5%, 5%, and 10%, with a carbon dioxide concentration of 5%. High-purity nitrogen was utilized as a balance gas to ensure appropriate atmospheric conditions during the experiments. For normoxic cell culture, the carbon dioxide concentration was maintained at 5%, while the oxygen concentration was set to atmospheric levels of approximately 21%. The incubator temperature was set at 37.5 °C, and the humidity was maintained at saturation. During the 4-day cell culture process at various oxygen concentrations, the initial cell density was set at 1 × 10^5^ cells per well in a 12-well cell culture plate. Daily cell counts were conducted using a cell counting chamber, and bright-field images of the cells were captured via the EVOS M5000 imaging system (Invitrogen, Carlsbad, CA, USA). Three biological replicates were performed for each test indicator to ensure data quality.

### 2.3. Induction of bSCs Meiosis In Vitro

To validate the germ cell characteristics of the isolated and cultured bSCs, we referenced previous experimental protocols [38,39] for the in vitro induction of bSCs meiosis. In this study, we added 1 μM of retinoic acid (Solarbio, Beijing, China, IR0060) and 1 μM of testosterone (Solarbio, IT0110) to the culture medium to induce meiosis in cultured bSCs for 20 days, after which the cells were collected for analysis.

### 2.4. Regulation of Glycolysis in bSCs

For analysis of the effects of lactate metabolic inhibition on cells, bSCs cultured with 5% oxygen were treated with 10 mM of sodium dichloroacetate (DCA) (ApexBIO, Houston, TX, USA, B7174) for 48 h [40], after which the cells were collected for subsequent experiments. For determination of the effects of inhibiting the expression of hexokinase 2 (HK-2) on glycolysis, histone lactylation, and the expression of proliferation-related genes in bSCs, the buffalo HK-2 gene interference siRNA product was obtained from Cas9x Biotechnology (order number: RY24001904). According to the protocol, the siRNA fragments were transfected into bSCs via Lipofectamine 3000 (Invitrogen, Carlsbad, CA, USA, L3000015), and a negative control group was established to demonstrate the specificity of the siRNA action. After transfection, the cells were cultured for 48 h and then collected for experimental analysis.

### 2.5. Immunofluorescence Analysis

Different groups of cells were collected and washed with PBS to remove culture medium components. At least three biological replicates were performed for each test indicator to ensure data quality. The cells were fixed in 4% paraformaldehyde for 30 min, followed by permeabilization with 0.4% Triton X-100. After washing, nonspecific binding sites were blocked with a blocking solution containing 1% BSA. The cells were incubated with a diluted primary antibody (Table 1) overnight at 4 °C and then washed to remove unbound antibodies. For the negative control samples, the blocking solution was used as a substitute during the primary antibody incubation step, while the secondary antibody and DNA staining procedures were identical to those of the other samples. After the samples were washed with PBS, they were incubated with a fluorescently labeled secondary antibody for one hour at room temperature in the dark, followed by additional washes. The nuclei were stained with the DNA-binding dye Hoechst 33342, washed again, and mounted on a glass slide with mounting medium. Finally, the stained cells were examined under a Nikon ECLIPSE Ts2-FL fluorescence microscope to visualize and capture images of the target antigens.

### 2.6. Western Blot Analysis

Proteins were extracted from cells via RIPA-PMSF lysis buffer (Solarbio, R0010), and the protein concentrations were determined for equal loading. Three biological replicates were performed for each test indicator to ensure data quality. The extracted proteins were then mixed with loading buffer and subjected to sodium dodecyl sulfate–polyacrylamide gel electrophoresis (SDS–PAGE), which separates the proteins on the basis of molecular weight. Following electrophoresis, the proteins were transferred from the gel to a nitrocellulose membrane (Solarbio, YA1711) by electroblotting (Tanon, Shanghai, China, VE-386). The membranes were incubated with a nonfat powdered milk blocking solution (Solarbio, D8340). The membranes were then incubated with a primary antibody specific to the target protein (Table 1) overnight at 4 °C, followed by washing to remove unbound antibodies with TBST (Solarbio, T1085). The secondary antibodies were added to the membranes to bind to the primary antibodies for one hour at room temperature; the membranes were further washed with TBST. After the membrane was soaked in enhanced chemiluminescence (ECL) substrate (Tanon, 180-506) for visualization, the Western blots were imaged via an E-Blot Touch Imager. The integrated optical density values of the bands for each sample were subsequently calculated via ImageJ 1.46 software. β-actin or histone H3 was used as a loading control to normalize the data, allowing the determination of the relative expression levels of the target protein across different samples for quantitative analysis.

### 2.7. Quantitative Real-Time PCR (qPCR) Analysis

Total RNA from different groups of bSCs was extracted with a TaKaRa MiniBEST Universal RNA Extraction Kit (TaKaRa, Tokyo, Japan, 9767). Three biological replicates were performed for each test indicator to ensure data quality. After the cells were lysed with the lysis buffer provided in the kit, they were placed on a spin column for separation, washed, and underwent other operations, and DEPC-treated water was used to elute the total RNA. The RNA concentration and purity were measured via a Thermo Scientific™ NanoDrop™ One microvolume UV–Vis spectrophotometer. The total RNA samples used for gene mRNA expression detection were grouped and reverse transcribed to obtain double-stranded cDNA according to the requirements of the PrimeScript™ RT Master Mix (TaKaRa, RR036). The qPCR system was then prepared according to the instructions of the TB Green^®^ Fast qPCR Mix (TaKaRa, RR430) kit. According to the reference sequences in GenBank, primers were designed via NCBl Primer Blast (Table 2). The primers were synthesized by Beijing Qingke Biotechnology Co., Ltd. The fluorescence signal was collected and analyzed with a Roche Light Cycler 480, and the relative expression of each target gene was calculated via the 2^−ΔΔCt^ method. β-actin was used as an internal reference gene, and cDNA was used as a template.

### 2.8. mRNA Library Construction and Next-Generation Sequencing (RNA-Seq) Analysis

Total RNA was extracted from cells cultured in 21% oxygen (No) or 5% oxygen (Hy), three biological replicates were sent for each group of cells for testing, and poly(A) selection was performed to enrich for mRNA, which was then fragmented and converted into complementary DNA (cDNA). This cDNA underwent end repair, adapter ligation, and amplification to create a sequencing library, and its quality was assessed via a Qubit instrument. The prepared library was sequenced on the DNBSEQ platform, which utilizes DNA nanoball technology for high-throughput sequencing, typically employing paired-end reads for increased accuracy. Following sequencing, the raw data were processed for quality control and alignment to a reference genome (NCBI, Bethesda, MD, USA, GCF_019923935.1_NDDB_SH_1) via alignment software. Differential gene expression analysis was conducted with tools such as DESeq2 to identify significantly regulated genes, while pathway enrichment analysis via Gene Ontology (GO) and Kyoto Encyclopedia of Genes and Genomes (KEGG) analyses elucidated the biological importance of these genes and their involvement in specific signaling pathways. This comprehensive approach enables the generation of high-quality transcriptomic data and provides insights into gene expression dynamics and associated biological processes. During data processing and visualization, we used the Sangerbox tool to complete the analysis [41].

### 2.9. Flow Cytometric Analysis

Three biological replicates were performed for each test indicator to ensure data quality. For analysis of DNA ploidy via propidium iodide (PI) staining [42] (PI/RNase Staining Buffer, BD Biosciences, Franklin Lakes, NJ, USA, 550825), the cells were first fixed in 70% ethanol for 30 min to 2 h, followed by washing with phosphate-buffered saline (PBS) and treatment with ribonuclease (RNase) to eliminate RNA. PI was then added to the cell suspension, and after a 30-min incubation at 37 °C, the samples were analyzed via a flow cytometer to quantify fluorescence intensity, allowing classification of cells into different ploidy categories on the basis of their DNA index (DI) values. For apoptosis detection via Annexin V-FITC/PI dual staining (PE Annexin V Apoptosis Detection Kit I, BD, Franklin Lakes, NJ, USA, 559763), the cells were prepared similarly and resuspended in binding buffer, followed by incubation with Annexin V-FITC and PI for 15 min at room temperature in the dark. These dual stains identify early apoptotic cells (annexin V-positive, PI-negative) and late apoptotic or necrotic cells (annexin V-positive, PI-positive). The samples were analyzed immediately by flow cytometry (Cytoflew S, Beckman Coulter, Indianapolis, IN, USA,) to determine the proportions of viable, early apoptotic, and late apoptotic/necrotic cells.

### 2.10. Immunohistochemistry (IHC) Analysis

We performed IHC-P staining on the testes of three 2-year-old buffaloes and five 5-month-old buffaloes. Testicular tissues were collected and fixed in 4% paraformaldehyde, followed by embedding in paraffin to prepare thin sections. Antigen retrieval was performed to unmask epitopes via heat-induced epitope retrieval with EDTA antigen retrieval solution (Solarbio, C1033). The sections were then incubated with a blocking solution to prevent nonspecific antibody binding before primary antibodies specific to the target proteins were applied (Table 1) overnight at 4 °C. After PBS washes, the sections were incubated with species-specific secondary antibodies conjugated to a reporter enzyme for one hour at room temperature, followed by additional washes. The chromogenic substrate c (Solarbio, DA1015) was applied, producing a brown precipitate at the site of protein expression, with hematoxylin counterstaining used to visualize the cell nuclei. Finally, the stained sections were examined under a Nikon ECLIPSE Ts2-FL microscope to assess protein localization and expression levels, allowing comparative analysis between the two age groups and providing insights into developmental changes in buffalo testes.

### 2.11. Lactate Level Analysis

We measured the lactate levels in various groups of bSCs via an Abbkine lactate detection kit (catalog no. KTB1100) following the operational steps outlined in the kit’s instructions. Three biological replicates were performed for each test indicator to ensure data quality. After the absorbance was measured with a BioTek Epoch microplate reader, we calculated the lactate concentration in each cell sample on the basis of a standard curve.

### 2.12. CUT&Tag-Seq Analysis

Different groups of cells of interest were collected and fixed to preserve chromatin structure; two biological replicates were sent for each group of cells for testing. Nuclei were isolated from the fixed cells through a lysis process that ensured the integrity of chromatin. The isolated nuclei were then subjected to tagmentation via Tn5 transposase, which simultaneously fragmented the DNA and added sequencing adapters. After tagmentation, the resulting DNA fragments were purified and amplified via polymerase chain reaction to generate a sequencing library, and the quality of the library was assessed to ensure that it met the requirements for high-throughput sequencing. The prepared library was sequenced on an Illumina NovaSeq platform, which provided high data output and quality. The raw sequencing data were subjected to quality control to filter out low-quality reads and remove adapter sequences, with the cleaned reads aligned to a reference genome via the alignment software Bowtie2. Peak calling was performed to identify regions of H3K18la enrichment via specialized software designed for low-background chromatin profiling SEACR. For analysis of the relationship between H3K18la and gene expression, differential expression analysis was conducted on RNA-Seq data from the same samples or comparable datasets, and the DESeq2 tool was used to identify differentially expressed genes based on statistical thresholds (false discovery rate < 0.05). Furthermore, transcription factor-binding sites were assessed by integrating H3K18la data, and pathway enrichment analysis was performed via the GO and KEGG databases to elucidate the biological importance of the differentially expressed genes associated with H3K18la.

### 2.13. Statistical Analysis

The statistical software SPSS 25.0 was used to conduct one-way analysis of variance (one-way ANOVA) on the data, and the related-sample *t*-test method was applied for comparisons between two experimental groups. For comparisons involving three or more experimental groups, the *F*-test was utilized. If significant differences were identified through ANOVA, Duncan’s New Multiple Range Test was employed for multiple comparisons. The results are expressed as means ± standard deviations, and GraphPad Prism 8.0 software was utilized for image production. A significant difference between the two groups was considered when *p* < 0.05.

## 3. Results

### 3.1. Hypoxia Promotes the Proliferation of bSCs

**Figure 1:**
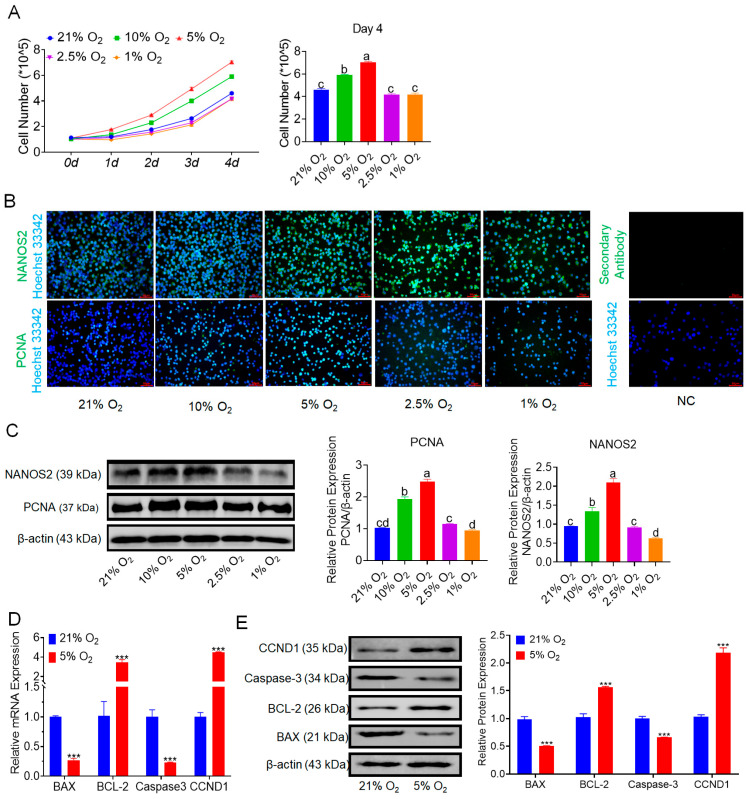
In vitro 5% oxygen culture conditions are conducive to increasing bSCs proliferation. (**A**): Growth curve of bSCs cultured under hypoxia and the results of cell number statistics on the fourth day; (**B**): Effects of different oxygen concentrations on the expression of NANOS2 and PCNA proteins in cultured bSCs (immunofluorescence staining); (**C**): Effects of different oxygen concentrations on the expression of NANOS2 and PCNA proteins in cultured bSCs (Western blotting, WB); (**D**): qPCR analysis of the effects of 5% and 21% oxygen concentrations on the mRNA levels of genes related to bSCs proliferation and apoptosis; (**E**): WB detection of the effects of 5% and 21% oxygen concentrations on the expression of proteins related to bSCs proliferation and apoptosis. [Abbreviations: NANOS2: nanos C2HC-type zinc finger 2. PCNA: proliferating cell nuclear antigen. BAX: Bcl-2-associated X protein. BCL-2: B-cell lymphoma 2. CCND1: cyclin D1. Magnification: 200×, scale bar = 50 μm; ***: *p* < 0.001; different letters represent significant differences between groups (*p* < 0.05)].

We cultured bSCs under various oxygen concentrations in vitro. Over a four-day period of monitoring cell counts, we found that the number of cells cultured under 5% oxygen was highest and significantly greater than those cultured under other oxygen concentrations (*p* < 0.05). Moreover, cell proliferation under 2.5% and 1% oxygen did not differ significantly from that under normoxia (*p* > 0.05) (Figure 1A). Brightfield images of the cells are presented in Figure S1A. We subsequently employed immunofluorescence staining techniques to evaluate and photograph the protein expression of NANOS2 (a key regulator involved in maintaining the undifferentiated state of spermatogonial cells) and PCNA (a marker of cell proliferation) across different groups of bSCs (Figure 1B and Figure S1B,C). Additionally, we conducted Western blot analysis and quantitative assessment of the expression of these two proteins. The results indicated that the expression levels of NANOS2 and PCNA in bSCs cultured under 5% oxygen conditions were significantly greater than those in the other groups (*p* < 0.05) (Figure 1C). We conducted transcriptomic sequencing on cells cultured under 21% and 5% oxygen conditions (Figure S1D–H). Through bioinformatic analysis, we performed enrichment analysis on differentially expressed genes related to cell proliferation regulation and generated a heatmap along with gene set enrichment analysis (GSEA) results (Figure S1I). We selected representative genes involved in apoptosis regulation, such as BAX, BCL-2, and Caspase-3, as well as genes regulating cell proliferation, such as CCND1, for qPCR and Western blot analysis. The results indicated that under 5% oxygen conditions, the expression of proapoptotic genes and proteins in bSCs significantly decreased (*p* < 0.05), whereas the expression of antiapoptotic and proliferation-promoting genes and proteins significantly increased (*p* < 0.05) (Figure 1D,E).

### 3.2. Hypoxia Benefits the Maintenance of bSCs

**Figure 2:**
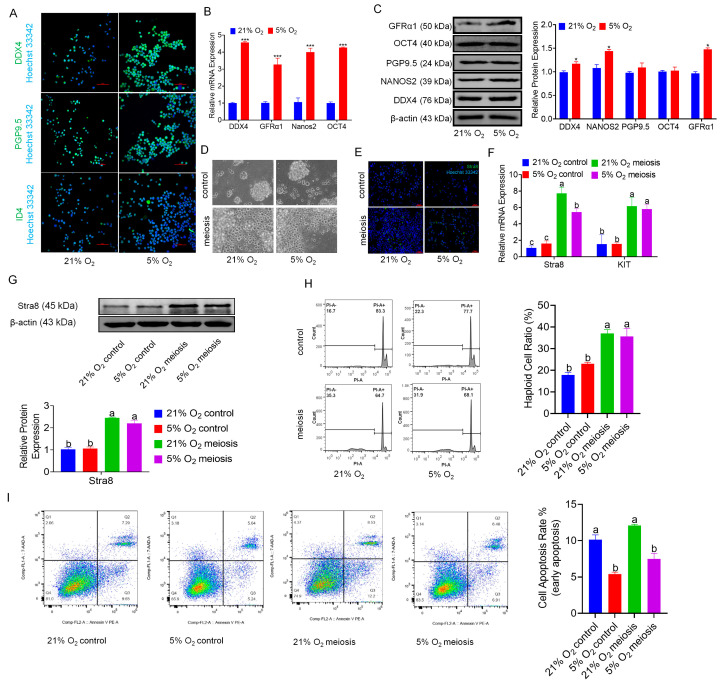
In vitro, 5% oxygen is conducive to maintaining the stem cell characteristics and reproductive characteristics of bSCs. (**A**): Effects of 5% and 21% oxygen concentrations on the expression of bSCs pluripotency (ID4) and reproductive specificity (PGP9.5, DDX4) proteins (immunofluorescence); (**B**): qPCR detection of the effects of 5% and 21% oxygen concentrations on the mRNA levels of bSCs pluripotency-related genes; (**C**): WB analysis of the effects of 5% and 21% oxygen concentrations on the expression of bSCs pluripotency-related proteins; (**D**): Cells were photographed under constant light to observe the effects of 5% and 21% oxygen concentrations on in vitro induced meiosis of bSCs; (**E**): Stra8 immunofluorescence staining was used to observe the effects of 5% and 21% oxygen concentrations on in vitro-induced meiosis of bSCs; (**F**): qPCR analysis of the effects of 5% and 21% oxygen concentrations on the mRNA levels of differentiation-related genes in the in vitro induced meiosis process of bSCs; (**G**): WB analysis of the effects of 5% and 21% oxygen concentrations on the expression of Stra8, a differentiation-related protein in the process of meiosis induced in vitro in bSCs; (**H**): Flow cytometric analysis of the effects of 5% and 21% oxygen on the ratio of nonpolyploid cells before and after meiosis in vitro in bSCs; (**I**): Flow cytometric analysis of the effect of 5% and 21% oxygen on the ratio of apoptosis of cells before and after meiosis in vitro in bSCs. [Abbreviations: PGP9.5: Protein Gene Product 9.5. ID4: Inhibitor of DNA Binding 4. GFRα1: Glial Cell Line-Derived Neurotrophic Factor Family Receptor Alpha 1. OCT4: Octamer-Binding Transcription Factor 4. DDX4: DEAD-Box Helicase 4. Stra8: Stimulated by Retinoic Acid Gene 8. KIT: KIT Proto-Oncogene, Receptor Tyrosine Kinase. Magnification: 200×, scale bar = 50 μm; *: *p* < 0.05; ***: *p* < 0.001; different letters represent significant differences between groups (*p* < 0.05)].

We conducted enrichment analysis on genes associated with stem cell self-renewal identified through transcriptomic sequencing, generating a heatmap and GSEA results (Figure S2A,B), which revealed an increasing trend of pluripotency-related gene expression in the hypoxia group. Immunofluorescence staining of the markers of stem cells and progenitor marker ID4 and the reproductive markers PGP9.5 and DDX4 in bSCs cultured under normoxic and hypoxic conditions demonstrated that both conditions effectively maintained stem cell and reproductive characteristics (Figure 2A and Figure S2C–E). Furthermore, the qPCR and Western blot results indicated that, compared with normoxic, bSCs cultured under hypoxia presented significant increases in pluripotency and reproductive characteristics (*p* < 0.05) (Figure 2B,C). To evaluate the impact of hypoxia on the meiotic function of bSCs, we induced meiosis in vitro via retinoic acid and testosterone under normoxic and hypoxic conditions and found a greater number of Stra8-positive cells in the meiosis induction group than in the control group, as shown by immunofluorescence staining (Figure 2D–E and Figure S2F). We assessed the mRNA levels of the Stra8 and KIT genes via qPCR and revealed that their expression was significantly greater in the meiosis induction group than in the control group (*p* < 0.05) (Figure 2F). Additionally, Western blot analysis revealed that the protein expression level of Stra8 was significantly greater in the meiosis induction group than in the control group (*p* < 0.05) (Figure 2G). Using flow cytometry, we assessed the DNA ploidy of bSCs and found that the proportion of nondiploid (non-2n/4n) cells was significantly greater in the meiosis induction group than in the control group (*p* < 0.05) (Figure 2H). To eliminate potential interference from apoptotic cells on the DNA ploidy results, we assessed apoptosis via flow cytometry, which revealed no significant differences in apoptosis levels between the control and meiosis induction groups under both normoxic (*p* > 0.05) and hypoxic conditions (*p* > 0.05) (Figure 2I). Interestingly, when the degree of apoptosis was compared between normoxic and hypoxic cultures, we found that hypoxic conditions significantly reduced the level of apoptosis in bSCs (*p* < 0.05), which is consistent with our previous findings.

### 3.3. Hypoxia Promotes Glycolysis and Lactate Accumulation in bSCs

**Figure 3:**
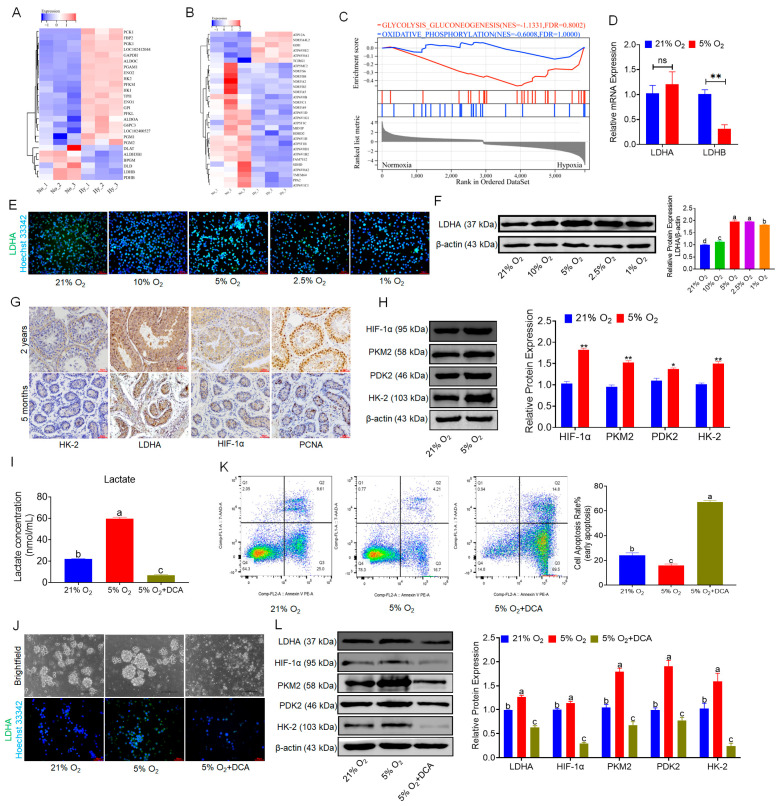
In vitro, 5% oxygen promoted glycolysis in bSCs and promoted lactate accumulation. (**A**): Heatmap of differentially expressed genes related to glycolysis in transcriptomic sequencing data; (**B**): Heatmap of differentially expressed genes related to oxidative phosphorylation in transcriptomic sequencing data; (**C**): GSEA of glycolysis and oxidative phosphorylation pathways; (**D**): qPCR results of LDHA and LDHB gene expression levels in bSCs cultured at 5% and 21% oxygen concentrations; (**E**,**F**): Immunofluorescence staining (**E**) and WB (**F**) results analyzing the effects of different oxygen concentrations on the key protein LDHA in bSCs glycolysis; (**G**): Immunohistochemistry analysis of the difference in glycolytic levels in the seminiferous tubules of adult and adolescent buffalo testes; (**H**): WB analysis of the effects of 5% and 21% oxygen concentrations on the expression of glycolysis-related proteins in bSCs; (**I**): After intracellular lactate metabolism was blocked with DCA, the changes in lactate levels of bSCs in each group were observed; (**J**): Brightfield analysis and LDHA fluorescence staining of the cells in each group; (**K**): Flow cytometric analysis of the effect of DCA blocking lactate metabolism on cell apoptosis; (**L**): WB analysis of the effect of DCA blocking lactate metabolism on the expression of proteins related to the glycolytic process. [Abbreviations: LDHA: Lactate Dehydrogenase A. LDHB: L-lactate Dehydrogenase B Chain. HK-2: Hexokinase II. HIF-1α: Hypoxia Inducible Factor 1 Subunit Alpha. PKM2: Pyruvate Kinase M2 Isoform. PDK2: Pyruvate Dehydrogenase Kinase Isoform 2. Magnification: 200×, scale bar = 50 μm; “ns” denotes no significant difference (*p* > 0.05); “*” indicates statistical significance (*p* < 0.05); “**” indicates highly significant difference (*p* < 0.01); different letters represent significant differences between groups (*p* < 0.05)].

To explore the mechanisms by which hypoxia promotes the proliferation of bSCs and maintains their pluripotency and reproductive specificity, we explored this mechanism on the basis of transcriptomic data, which revealed that hypoxia alters cellular metabolic levels, leading to notable changes in glycolysis and oxidative phosphorylation-associated gene expression, as summarized in a heatmap and GSEA. Compared with that in the normoxic group, the glycolytic activity of bSCs in the hypoxic group was increased, whereas the level of oxidative phosphorylation was decreased (Figure 3A–C). The qPCR results revealed that, compared with that in the normoxia group, the LDHA expression in bSCs in the hypoxia group was not significantly different (*p* > 0.05), but LDHB was significantly downregulated in the hypoxia group (*p* < 0.05) (Figure 3D). Using immunofluorescence staining and Western blot techniques, we qualitatively and quantitatively analyzed LDHA protein expression in bSCs cultured at various oxygen concentrations and revealed that LDHA expression increased as the oxygen concentration decreased, with a significant increase in LDHA expression in cells cultured under 5% oxygen compared with those cultured under normoxic conditions (*p* < 0.05) (Figure 3E,F and Figure S3A). We conducted immunohistochemical analysis on testicular tissues from sexually mature adult buffalo (2 years old) and prepubescent buffalo (5 months old) to evaluate the expression and localization of the key glycolytic enzymes HK-2 and LDHA, the hypoxia-inducible factor HIF-1α, and the cell proliferation regulator PCNA, revealing that glycolysis-active cells were predominantly localized near the basement membrane of the seminiferous tubules, which was consistent with high levels of hypoxia-inducible factors and increased proliferative activity, regardless of sexual maturity (Figure 3G). We performed Western blot analysis of glycolysis-related proteins (HIF-1α, PKM2, PDK2, and HK-2) in bSCs cultured under hypoxic and normoxic conditions and found that their expression levels were significantly greater in the hypoxic group than in the normoxic group (*p* < 0.05) (Figure 3H). To confirm the role of glycolysis in hypoxia-mediated promotion of bSCs proliferation, we used DCA. This treatment significantly decreased intracellular lactate levels in bSCs cultured under both hypoxic and normoxic conditions (*p* < 0.05) (Figure 3I). Immunofluorescence staining was also used to determine the expression of LDHA in different groups of cells (Figure 3J and Figure S3B). Flow cytometric analysis of the degree of apoptosis across the groups revealed that, compared with those of both the normoxia and hypoxia groups, the addition of DCA during hypoxia culture resulted in a significant increase in the degree of cell apoptosis (*p* < 0.05) (Figure 3K). Furthermore, Western blot analysis of glycolysis-related protein expression changes revealed that, following DCA treatment to inhibit glycolysis, the expression levels of glycolysis-related proteins (LDHA, HIF-1α, PKM2, PDK2, and HK-2) were significantly lower than those in both normoxic and hypoxic cultures (*p* < 0.05) (Figure 3L).

### 3.4. Hypoxia Elevates H3K18 Lactylation in bSCs

**Figure 4:**
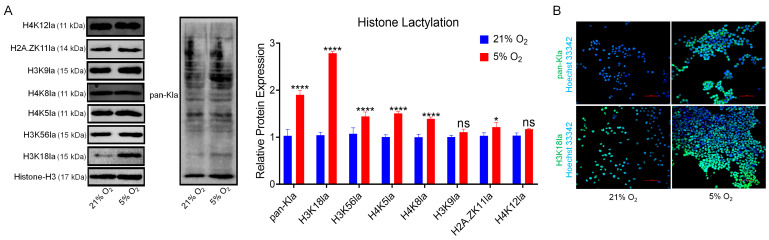
In vitro, 5% oxygen promoted an increase in H3K18la histone lactic acid modification in bSCs. (**A**): WB analysis of the effects of 5% and 21% oxygen concentrations on the lactic acid modification of histones and total proteins in bSCs; (**B**): Immunofluorescence analysis of the effects of 5% and 21% oxygen concentrations on lactic acid-modified total proteins and H3K18la proteins in bSCs. [Abbreviations: H4K12la: Histone H4 lysine 12 lactylation. H2A.ZK11la: Histone H2A variant Z lysine 11 lactylation. H3K9la: Histone H3 lysine 9 lactylation. H4K8la: Histone H4 lysine 8 lactylation. H4K5la: Histone H4 lysine 5 lactylation. H3K56la: Histone H3 lysine 56 lactylation. H3K18la: Histone H3 lysine 18 lactylation. pan-Kla: Pan-histone lysine lactylation. Magnification: 200×, scale bar = 50 μm; “ns” denotes no significant difference (*p* > 0.05); “*” indicates statistical significance (*p* < 0.05); “****” indicates extremely significant difference (*p* < 0.0001); magnification: 200×, scale bar = 50 μm].

We employed Western blot analysis to assess the expression levels of common lactylation-modified histones (H4K12la, H2A. ZK11la, H3K9la, H4K8la, H4K5la, H3K56la, and H3K18la) and pan-lactylated proteins (pan-Kla) in bSCs cultured under normoxia and hypoxia, revealing that most lactylation levels were significantly increased in hypoxia compared with normoxia, with the most striking difference observed in H3K18la (*p* < 0.05) (Figure 4A). Furthermore, immunofluorescence staining of pan-Kla and H3K18la in bSCs cultured under normoxia and hypoxia confirmed their expression in bSCs (Figure 4B and Figure S4A,B).

### 3.5. Combined CUT&Tag-Seq and RNA-Seq Identified the Target Genes That Promote bSCs Proliferation Through H3K18la

**Figure 5:**
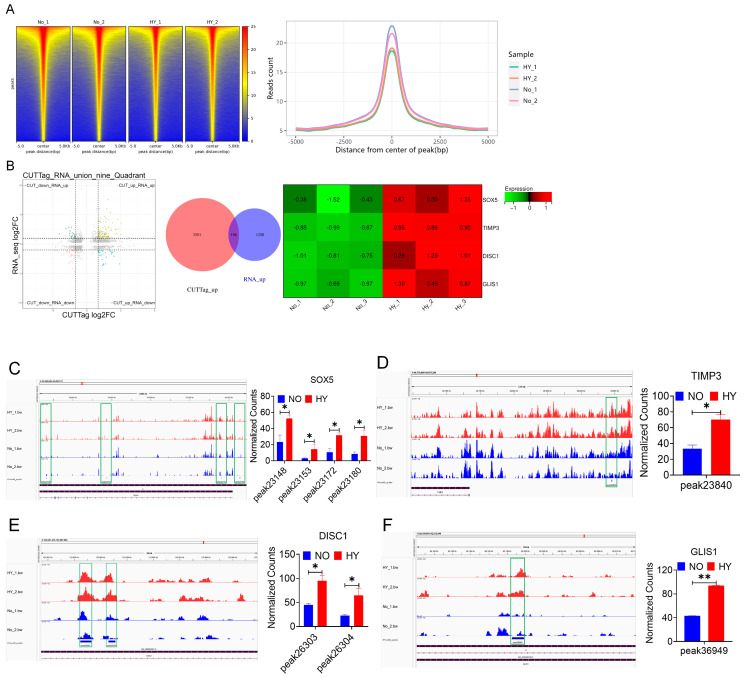
CUT&TAG-seq combined with RNA-seq technology revealed that a 5% oxygen concentration in vitro promoted the proliferation of bSCs in vitro by activating H3K18la lactic acid expression. (**A**): Signal heatmap near the center of each group peak and signal distribution map near the center of the peak; (**B**): Nine-quadrant map of CUT&RNA joint analysis, up-up Venn diagram and RNA-seq screening heatmap of differentially expressed genes; (**C**–**F**): Integrative Genomics Viewer (IGV) visualization results of each site of the enriched differentially expressed genes SOX5 (**C**), TIMP3 (**D**), DISC1 (**E**) and GLIS1 (**F**) and relative quantitative results of CUT&Tag enrichment normalized counts. [Abbreviations: SOX5: SRY-Box Transcription Factor 5. TIMP3: Tissue Inhibitor of Metalloproteinases 3. DISC1: Disrupted in Schizophrenia 1. GLIS1: GLIS Family Zinc Finger 1; “*” indicates statistical significance (*p* < 0.05); “**” indicates highly significant difference (*p* < 0.01); The green box in the figure represents the enrichment curve differences of differentially expressed sites visualized in the IGV image. It highlights regions where differential expression peaks are enriched, reflecting variations in gene regulation or chromatin features across samples].

To elucidate the role of H3K18la as an epigenetic modification in promoting the proliferation of bSCs under hypoxia, we employed CUT&Tag-seq technology to identify genes or transcription factors enriched with H3K18la (Figure S5A–C). Figure 5A displays heatmaps and signal distribution plots near the peak centers for each group. We subsequently integrated the transcriptomic sequencing data with CUT&Tag-seq data to perform a joint analysis. Through functional exploration of the jointly upregulated differentially expressed genes, we identified four key genes associated with the maintenance of pluripotency and proliferative activity: SOX5, TIMP3, DISC1, and GLIS1 (Figure 5B and Figure S5D). We visualized the differential peak distributions and counts for the four enriched genes in bSCs, revealing that, compared with normoxia, hypoxia significantly increased the enrichment of H3K18la in genes related to pluripotency maintenance and proliferation (*p* < 0.05) (Figure 5C–F).

### 3.6. HK-2 Knockdown Inhibits Glycolysis and Proliferation in bSCs

**Figure 6:**
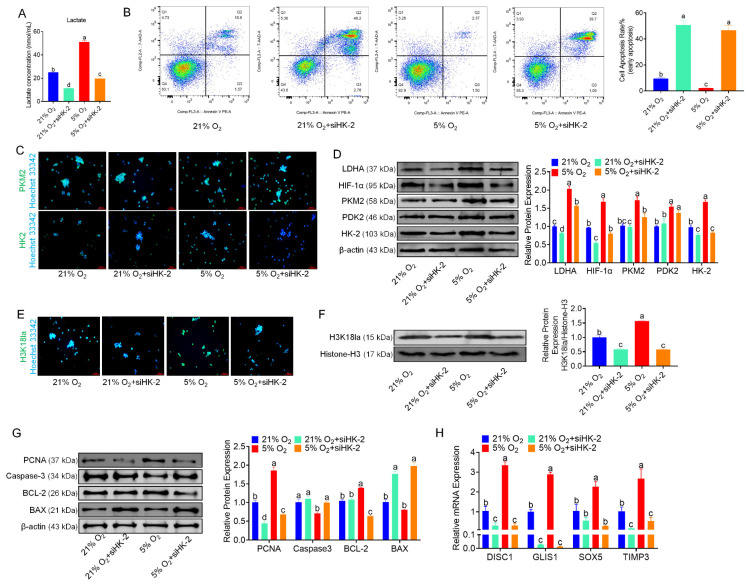
Inhibition of HK-2 expression inhibits glycolysis and interferes with the effect of hypoxia on the proliferation of bSCs in vitro. (**A**): After the HK-2 gene was silenced via RNAi, changes in the lactate levels of bSCs in each group were observed. (**B**): Flow cytometric analysis of the effect of inhibiting HK-2 expression on cell apoptosis. (**C**): Immunofluorescence staining was used to analyze the effect of inhibiting HK-2 expression on key proteins involved in glycolysis in bSCs. (**D**): WB analysis of the effect of inhibiting HK-2 expression on key proteins involved in glycolysis in bSCs. (**E**): Immunofluorescence staining was used to analyze the effect of inhibiting HK-2 expression on H3K18la protein at the lactic acid modification site of bSCs. (**F**): WB analysis was used to analyze the effect of inhibiting HK-2 expression on H3K18la protein at the lactic acid modification site of bSCs. (**G**): WB analysis was used to analyze the effect of inhibiting HK-2 expression on phenotypic proteins related to the proliferation and apoptosis of bSCs. (**H**): qPCR detection was used to analyze the effect of inhibiting HK-2 expression on SOX5, TIMP3, DISC1, and GLIS1, target genes associated with lactic acid modification of H3K18la in bSCs. [Magnification: 200×, scale bar = 50 μm; different letters represent significant differences between groups (*p* < 0.05)].

We utilized RNA interference (RNAi) technology to inhibit the expression of HK-2 in bSCs cultured under both normoxia and hypoxia, selecting the most effective interference site for subsequent experiments (Figure S6A). The measurement of lactate levels in each group revealed that after HK-2 expression was inhibited, the lactate levels in the siHK-2 group were significantly lower than those in the control group under both normoxia and hypoxia (*p* < 0.05) (Figure 6A). Flow cytometric analysis of the degree of apoptosis across the groups revealed that, following the inhibition of HK-2 expression in bSCs cultured under both normoxia and hypoxia, the cell apoptosis rates were significantly higher in the siHK-2 group than in the control group (*p* < 0.05) (Figure 6B). Immunofluorescence staining was employed to observe the expression of key proteins involved in the glycolytic pathway within the cells (Figure 6C and Figure S6B–F). Additionally, Western blot analysis revealed that following the inhibition of HK-2 expression in bSCs cultured under both normoxia and hypoxia, the expression levels of LDHA, HIF-1α, and HK-2 were significantly lower in the siHK-2 group than in the control group (*p* < 0.05). Notably, inhibiting HK-2 expression did not affect PDK2 levels (*p* > 0.05), while the suppression of HK-2 had a significant inhibitory effect on PKM2 expression only under hypoxia (*p* < 0.05) (Figure 6D). We investigated the impact of HK-2 expression inhibition on H3K18la through immunofluorescence staining and Western blot analysis and found that the H3K18la expression levels were significantly lower in the siHK-2 group than in the control group under both normoxic and hypoxic bSCs culture conditions (*p* < 0.05) (Figure 6E,F and Figure S6G). Additionally, we employed immunofluorescence staining to assess the expression of pluripotency- and reproductive specificity-related proteins in bSCs following HK-2 inhibition. The findings revealed that inhibiting HK-2 did not significantly affect the expression levels of DDX4, PGP9.5, ID4, or NANOS2 (Figure S6H–K). Furthermore, Western blot analysis of proteins associated with cell proliferation and apoptosis revealed that under both normoxia and hypoxia, the expression level of PCNA was significantly lower in the siHK-2 group than in the control group (*p* < 0.05). Conversely, BAX protein levels were significantly increased (*p* < 0.05). Under hypoxia, siHK-2 treatment resulted in a significant increase in Caspase-3 protein levels and a significant decrease in BCL-2 expression compared with those in the control group (*p* < 0.05), whereas inhibiting HK-2 expression did not significantly affect Caspase-3 and BCL-2 levels under normoxic conditions (*p* > 0.05) (Figure 6G). Through qPCR analysis, we investigated the changes in the expression of SOX5, TIMP3, DISC1, and GLIS1 in bSCs after HK-2 interference. Compared with those in the control group, the expression levels of SOX5, TIMP3, DISC1, and GLIS1 in the siHK-2 group were significantly lower under hypoxia (*p* < 0.05). Additionally, regardless of whether the cells were cultured under normoxia or hypoxia, the siHK-2 group presented significant downregulation of DISC1, GLIS1, and TIMP3 compared with the control group (*p* < 0.05). Notably, SOX5 expression was significantly inhibited by siHK-2 only under hypoxia, with no significant difference observed under normoxia (Figure 6H).

## 4. Discussion

Despite our previous research exploring the in vitro culture of SCs from water buffaloes and achieving successful long-term cultivation, these advancements are still insufficient for subsequent applications and studies [8,43,44]. The current methodologies need further refinement to increase the viability and functionality of SSCs over extended periods [9]. Current culture systems cannot fully mimic the physiological conditions found in vivo, which can lead to suboptimal growth and differentiation outcomes [10]. In vivo oxygen tensions vary significantly across tissues and developmental stages. Phosphorescence lifetime imaging in live mice revealed that bone marrow monocytes experience physiological oxygen levels of 5.3% under normoxic conditions, which decrease to 2.4% under hypoxia [45]. Renal tubular cells exhibit intracellular oxygen tensions ranging from 1% to 5% depending on metabolic demands [46]. Neural stem cells cultured at 2.5–5% oxygen show enhanced survival and differentiation compared to 20% [47]. These findings underscore the importance of tailoring in vitro oxygen levels to mimic tissue-specific microenvironments. Our findings demonstrate that culturing bSCs under 5% oxygen tension significantly enhances their proliferative capacity in vitro compared to atmospheric oxygen levels (21% O_2_). This aligns with emerging evidence that physiological oxygen microenvironments critically regulate stem cell behavior, a paradigm now validated across multiple mammalian systems [18,48]. Notably, our observation of reduced proliferation at ≤1% O_2_ mirrors studies in murine SSCs, where severe hypoxia (1% O_2_) impaired germ cell cluster formation and induced differentiation bias. The species-specific divergence in optimal oxygen thresholds (5% O_2_ for buffalo vs. 3.5–4% for mice) likely reflects evolutionary adaptations to distinct testicular niches, warranting further comparative analysis of hypoxic signaling pathways across bovids and rodents [18]. While our study identifies 5% O_2_ as optimal for bSC proliferation, this contrasts with findings in human mesenchymal stem cells (hMSCs) [49]. This discrepancy underscores the importance of tissue-specific oxygen optimization: testicular stem cells, evolutionarily adapted to chronic hypoxia (<5% O_2_ in seminiferous tubules), may require higher oxygen thresholds than mesenchymal lineages [50]. This emphasizes the critical need to redefine “normoxia” in cell culture systems based on tissue-specific physiology rather than atmospheric standards. Our study focuses on promoting spermatogonial cells proliferation in vitro by mimicking the in vivo moderate hypoxic niche, which has been shown to enhance stem cell maintenance, while recognizing that severe hypoxia can be detrimental [18,51]. Stem cells, including spermatogonial, neural, and embryonic stem cells, adapt to hypoxic niches by activating HIF-mediated pathways that promote metabolic reprogramming toward glycolysis and engage signaling networks such as Wnt/β-catenin and Notch to maintain pluripotency and drive proliferation [52]. In contrast, differentiated cells typically respond to hypoxia with cell cycle arrest mediated by HIF-induced expression of cell cycle inhibitors [53]. Although the effects of hypoxia on other testicular cells like Sertoli and Leydig cells are complex and not addressed here, future work using co-culture or organoid models will be essential to investigate cell-type specific responses within the heterogeneous and dynamic oxygen microenvironment of the testis [20,54,55]. We acknowledge these limitations and have included this discussion to guide further research on the broader impact of hypoxia in testicular biology. Our findings carry important translational implications; they suggest current SSC expansion protocols may be suboptimal due to oxygen mismatch.

The maintenance of stem cell pluripotency and self-renewal capabilities is critically dependent on recapitulating physiological oxygen microenvironments in vitro. Studies have shown that human embryonic stem cells (hESCs) cultured at physiological oxygen levels (approximately 5% O_2_) retain their stemness better than those cultured at atmospheric levels do, which leads to chromosomal instability [50]. The physiological oxygen tension found in the testicular niche is significantly lower than the atmospheric oxygen tension, indicating that current culture protocols may not adequately mimic the in vivo environment necessary for optimal SSC growth [56,57]. Our findings demonstrate that culturing bSCs under 5% oxygen tension significantly enhances the expression of pluripotency-associated genes and activates key signaling pathways compared to normoxic conditions. Our measurement of ~5% intra-testicular oxygen in water buffaloes positions this species within the broader mammalian paradigm, where SSC niches typically operate at 1–5% O_2_ [58]. Owing to experimental constraints, although we were unable to perform SSC transplantation experiments in water buffaloes for validation, our experiments with in vitro induction of meiosis further supported our hypothesis to the greatest extent possible. For translational applications—particularly in vitro gametogenesis—implementing physiologically-relevant oxygen tensions could enhance germ cell yield while maintaining genetic fidelity.

The metabolic implications of hypoxia are particularly salient. At 5% O_2_, bSCs exhibited enhanced glycolytic flux and lactate accumulation—a phenomenon corroborated by recent work showing that physiological hypoxia (2–8% O_2_) triggers metabolic reprogramming in human fibroblasts and adipocytes [59]. By mining transcriptomic sequencing data for pathways and genes that are highly associated with glycolysis and hypoxia, we confirmed that alterations in metabolic pathways are the primary mechanism by which hypoxia promotes the proliferative activity of bSCs. Under hypoxic conditions, cells often switch from oxidative phosphorylation to glycolysis for energy production. This shift is mediated by the activation of HIFs, which upregulate genes associated with glycolytic enzymes. While lactate accumulation is typically viewed as a glycolytic waste product, our data and recent studies implicate it as a pleiotropic signaling molecule [60,61]. We hypothesize that, in vivo, the glycolytic levels of cells within the seminiferous tubules of the testicular tissue are also high, particularly for SSCs near the basal membrane of the seminiferous tubules. The results of the IHC-P staining performed on testicular tissue confirmed our hypothesis. Research has shown that undifferentiated spermatogonial cells exhibit a greater glycolytic capacity than differentiating spermatogonial cells do, suggesting that high levels of glycolysis are critical for maintaining the proliferative activity of SSCs [62]. The metabolic pathways ensure that sufficient energy is available for cell division and differentiation processes [63]. DCA’s ability to reverse hypoxia-induced glycolytic shifts in cardiac tissue [64] contrasts sharply with its pro-apoptotic effects in testicular models. This divergence underscores the tissue-specificity of metabolic adaptations—while cardiac myocytes benefit from restored oxidative metabolism, SSCs reliant on glycolysis undergo catastrophic energy failure. These experimental results indicate that the ability of hypoxia to promote cell proliferation is indeed caused by its activation of glycolysis.

Culturing cells under hypoxic conditions promotes a metabolic shift toward increased glycolysis while reducing oxidative phosphorylation [26]. This metabolic adaptation is crucial for maintaining energy production without the detrimental effects associated with high oxygen levels, which can lead to increased ROS production and cellular senescence [65]. Hypoxia can induce epigenetic changes such as histone modifications that increase the expression of genes related to growth and survival, thereby further promoting cellular proliferation under low-oxygen conditions [66]. Lactate, a byproduct of glycolysis, has been identified as a substrate for histone lactylation, a novel epigenetic modification [35,36]. Studies have shown that high glycolytic flux is essential for preserving the undifferentiated state of SSCs, particularly in culture conditions that mimic their natural niche [67]. Increased levels of lactate, often resulting from increased glycolytic activity, can lead to increased histone lactylation. This modification may affect the transcriptional landscape of SSCs by promoting the expression of genes associated with stemness while repressing those related to differentiation [68]. Studies have shown that Dux-induced H3K18 lactylation plays a crucial role in increasing the efficacy of induced iPSC reprogramming by controlling the metabolism-H3K18la-MET network, with specific recruitment of the protein Brg1 to promoters of pluripotency-related genes [34]. Our results indicated that there was a significant difference in histone H3K18la levels between bSCs cultured under normoxic and hypoxic conditions. Crucially, our data extend these observations to ruminant germline stem cells, demonstrating conserved metabolic plasticity across taxa. The inverse correlation between oxygen tension and H3K18la lactylation levels suggests histone lactylation may serve as an epigenetic rheostat coordinating hypoxia-induced proliferation—a mechanism recently implicated in cancer stem cell maintenance.

Our findings demonstrate that hypoxia-induced H3K18la drives bSCs proliferation by activating transcriptional programs associated with stemness maintenance and metabolic plasticity. Through integrated CUT&Tag, transcriptomic, and functional analyses, we identified SOX5, GLIS1, TIMP3, and DISC1 as key mediators of lactylation-dependent proliferation—a mechanism distinct from canonical HIF pathways while retaining evolutionary conservation with other stem cell systems. Under hypoxic conditions, bSCs exhibit an increase in intracellular lactate, mirroring lactate accumulation patterns observed in tumor microenvironments [69] and neuroectodermal differentiation models [70]. This metabolic shift correlates with elevated H3K18la levels at promoter/enhancer regions of stemness-associated genes as demonstrated by CUT&Tag-seq. SOX5 may increase cell proliferation by promoting the expression of genes associated with stemness and inhibiting differentiation signals [71]. Known for its role in extracellular matrix remodeling, TIMP3 can influence cell proliferation and survival [72]. DISC1 may play a role in promoting proliferation through its involvement in neurodevelopmental signaling pathways or by modulating cellular responses to environmental cues [73]. GLIS1 can promote the maintenance of stem cell characteristics, thus supporting their proliferation by activating genes necessary for sustaining an undifferentiated state [74]. The discovery that hypoxia-induced H3K18la drives bSC proliferation through SOX5, GLIS1, TIMP3, and DISC1 activation represents a paradigm shift in understanding germline metabolic regulation. To verify whether the identified genes are key targets for hypoxia-induced proliferation of bSCs, we utilized RNAi to knock down the expression of HK-2, a critical rate-limiting enzyme upstream of the glycolysis pathway [75]. This approach allowed us to observe changes in lactate and H3K18la expression levels, as well as the expression of the four target genes (SOX5, TIMP3, DISC1, and GLIS1), after blocking the glycolytic pathway. These results indicate that inhibiting HK-2 expression leads to significant decreases in lactate levels and glycolytic activity in bSCs, along with a marked reduction in H3K18la levels. This inhibition also results in the downregulation of genes associated with cell proliferation and the upregulation of apoptosis-related genes, as well as the significant downregulation of the four key target genes (Figure 6). These results indicate that the increased proliferation of bSCs under hypoxic conditions may be attributed to elevated lactate levels resulting from increased glycolytic activity, which in turn leads to increased H3K18 lactylation. This modification subsequently regulates the upregulation of key transcription factors, including SOX5, TIMP3, DISC1, and GLIS1, ultimately promoting cell proliferation in vitro. Our work repositions lactate from a passive glycolysis byproduct to a central regulator of germline epigenetics. These mechanisms collectively address a fundamental paradox in male fertility—maintaining active spermatogenesis in naturally hypoxic testes. By integrating findings from cancer biology [76,77], immunology [78], and developmental genetics [35], we establish H3K18la as a linchpin of metabolic-epigenetic crosstalk with broad implications for regenerative medicine and oncogenesis.

Our results confirmed that culturing bSCs under low oxygen concentration (approximately 5%) significantly increased cell proliferation without impairing stem cell properties or reproductive functions. Under hypoxic conditions, glycolysis in bSCs is activated, leading to a significant increase in lactate production, which in turn promotes histone lactylation, particularly at H3K18la. By utilizing CUT&Tag-seq and RNA-seq for a combined analysis of gene expression associated with H3K18la, we elucidated the effects of normoxia and hypoxia on its expression and revealed that the mRNA levels and H3K18la-associated expression of SOX5, TIMP3, DISC1, and GLIS1 were significantly elevated during hypoxia, with these genes being related primarily to cell proliferation and stem cell characteristics. Furthermore, by inhibiting the expression of HK-2, the effects of hypoxia on promoting cell proliferation, H3K18la expression, and the expression of these four genes were neutralized. Our study demonstrates that hypoxia-induced glycolysis and H3K18la lactylation synergistically promote spermatogonial cells proliferation, laying a foundation for enhancing in vitro culture systems and breeding strategies in buffalo and other livestock [18,62]. Additionally, these findings provide important insights into the epigenetic regulation of SSCs, which may inform the development of novel therapies for male infertility and advance stem cell–based reproductive medicine in humans [35,79,80]. However, the primarily in vitro nature of our experiments and the species-specific focus limit the generalizability and physiological relevance of our results. Therefore, further in vivo studies and detailed mechanistic investigations are necessary to fully elucidate the underlying molecular pathways and translate these findings into broader practical applications.

## 5. Conclusions

These results suggest that the mechanism by which hypoxia promotes bSCs proliferation involves enhanced glycolysis, leading to elevated lactate production. The increased lactate subsequently promotes histone lactylation, particularly at H3K18la, which alters the expression of its target genes such as SOX5, TIMP3, DISC1, and GLIS1, ultimately resulting in increased cell proliferation.

## Figures and Tables

**Table 1:** Antibody.

Protein Name	Manufacture	Catalog Number	IF Dilution	WB Dilution	IHC-P Dilution
NANOS2	Santa Cruz (Dallas, Texas, USA)	sc-393794	1:100	1:1000	-
PCNA	Proteintech (Wuhan, China)	10205-2-AP	1:500	1:5000	1:1000
CCND1	HUABIO (Hangzhou, China)	ET1601-31	-	1:1000	-
Caspase-3	Abcam (Cambridge, UK)	ab13847	-	1:1000	-
BCL-2	Proteintech	68103-1-Ig	-	1:1000	-
BAX	Proteintech	50599-2-Ig	-	1:1000	-
ID4	Abcam	ab220881	1:200	-	-
PGP9.5	Abcam	ab108986	1:200	1:1000	-
DDX4	Abcam	ab13840	1:200	1:1000	-
GFRα1	Abcam	ab186855	-	1:1000	-
OCT4	Abcam	ab181557	-	1:1000	-
Stra8	Bioss (Beijing, China)	bs-1903R	1:100	1: 1000	-
LDHA	Bioss	bs-1810R	1:100	1:1000	1:100
HIF1a	ZenBio (Chengdu, China)	340462	1:100	1: 500	1:100
PKM2	ZenBio	321004	1:100	1: 500	-
PDK2	ZenBio	R382183	1:100	1: 500	-
HK-2	ZenBio	R24552	1:100	1: 500	1:100
H4K12la	PTMbio (Hangzhou, China)	PTM-1411RM	-	1:1000	-
H2A.ZK11la	PTMbio	PTM-1422RM	-	1:1000	-
H3K9la	PTMbio	PTM-1419RM	-	1:1000	-
H4K8la	PTMbio	PTM-1415RM	-	1:1000	-
H4K5la	PTMbio	PTM-1407RM	-	1:1000	-
H3K56la	PTMbio	PTM-1421RM	-	1:1000	-
H3K18la	PTMbio	PTM-1427RM	1:100	1:1000	-
Pan-Kla	PTMbio	PTM-1401RM	1:100	1:1000	-
β-actin	Servicebio (Wuhan, China)	GB11001	-	1:4000	-
Histone-H3	Proteintech	17168-1-AP	-	1:2000	-
HRP conjugated Goat Anti-Rabbit	Servicebio	GB23303	-	1:10,000	1:1000
HRP conjugated Goat Anti-Mouse	Servicebio	GB23301	-	1:10,000	1:1000
CoraLite488-conjugated Goat Anti-Mouse	Proteintech	SA00013-1	1:1000	-	-
CoraLite488-conjugated Goat Anti-Rabbit	Proteintech	SA00013-2	1:1000	-	-

**Table 2:** Primer sequences.

Genes		Primer Sequences (5′-3′)	NCBI Reference Sequence
β-actin	Forward	GATGATGATATTGCCGCGCTC	NM_001290932.1
	Reverse	CCGTGCTCAATGGGGTACTT	
BAX	Forward	CCAGAGGCGGGGTTTCAT	XM_025269476.3
	Reverse	CAGCTGCGATCATCCTCTGT	
BCL-2	Forward	CTTCCGTTTGCTCGTGCTCT	XM_025273635.3
	Reverse	ACCTCCTCCGTGATGTTGTA	
Caspase-3	Forward	TGGAACCAATGGACCTGTCG	XM_025280224.2
	Reverse	CCAGGATCCGTACTTTGCGT	
CCND1	Forward	GCCGAGGAGAACAAGCAGAT	XM_006072357.4
	Reverse	TGTCAGGCGGTGATAGGAGA	
DDX4	Forward	AAGCCGCTGAGAGGTACAAC	NM_001319794.1
	Reverse	TTCTGACGATGAAGCCGGAG	
GFRα1	Forward	GTGGGCGTCCCTGAACTTT	XM_044934905.2
	Reverse	CGGTTCCGCTTTTAGGGGTT	
Nanos2	Forward	CCACCTACCACCAACACTCC	XM_025269353.3
	Reverse	TGCAAAAGTTGCACAGGGTG	
OCT4	Forward	TGGGAAACCTTTGACCCACC	XM_006052299.4
	Reverse	TTCCGGCTATGGTGAGGGTA	
STRA8	Forward	CAGAGTTCGTGAGCAGAGGC	XM_044946902.1
	Reverse	CCCTGCCACAGCCATATTTC	
KIT	Forward	AGGTGTTCTGTTCCCGTTGG	NM_001290952.1
	Reverse	GACATGGATTGTTCTTTAAATGC	
HK-2	Forward	CAGGAGATCGACATGGGCTC	XM_006067902.4
	Reverse	GATCTGGCAGACCCGATGAG	
DISC1	Forward	TCAACTGCACATGGCAATCC	XM_025284501.2
	Reverse	CACTCCTCCCTCCATCCTGT	
GLIS1	Forward	TCCCCTATGGTGAGTGCTAC	XM_025288700.2
	Reverse	AAGGTGCACTAAGGCCATGA	
SOX5	Forward	CCAGGTGATGCTTCCAGGAC	XM_045165614.1
	Reverse	GCCAACAACACAAAGGCTCA	
TIMP3	Forward	GGACCCGCGCTATATCACTC	XM_006069554.4
	Reverse	TGCTGCTCGAGTCTCCAAAG	
LDHA	Forward	CTCGCGGTTCCATTTAAGGC	XM_025286309.3
	Reverse	AGCTGATCCTTGAGAGTTGCC	
LDHB	Forward	AGTGGATTACCCAAGCACCG	XM_006067641.4
	Reverse	TCCCCCAAAATCCATCCGTG	

## Data Availability

The original contributions presented in this study are included in the article/supplementary material. Further inquiries can be directed to the corresponding authors.

## Supplementary Materials

The following supporting information can be downloaded at: https://www.mdpi.com/article/10.3390/cells14110832/s1, Figure S1: Effects of Oxygen Concentration on bSCs: Morphology, Protein Expression, Gene Correlation, Differential Expression, and Pathway Analysis; Figure S2: Transcriptomic and Immunofluorescence Analysis of Pluripotency and Meiosis-Related Proteins in bSCs under Normoxia and Hypoxia; Figure S3: Multichannel immunofluorescence staining images; Figure S4: Multichannel Immunofluorescence of pan-Kla and H3K18la Protein Localization in bSCs under Normoxia and Hypoxia; Figure S5: CUT&Tag and RNA-Seq Analysis: Correlation, Differential Peaks, Functional Annotation, and Pathway Enrichment; Figure S6: Effects of HK-2 Knockdown on Glycolytic Proteins, Histone Lactylation, and Stem Cell Markers in bSCs under Normoxia and Hypoxia.

## Abbreviations

The following abbreviations are used in this manuscript:

SSCs	Spermatogonial stem cells
ASC	Adult stem cell
iPSCs	Induced pluripotent stem cells
bSCs	Buffalo spermatogonial cells
FBS	Fetal bovine serum
DCA	Sodium dichloroacetate
WB	Western blot
SDS–PAGE	Sodium dodecyl sulfate–polyacrylamide gel electrophoresis
ECL	Enhanced chemiluminescence
qPCR	Quantitative real-time PCR
RNA-Seq	Next-generation sequencing
GO	Gene Ontology
KEGG	Kyoto Encyclopedia of Genes and Genomes
PI	Propidium iodide
PBS	Phosphate-buffered saline
IHC	Immunohistochemistry
hMSCs	Human mesenchymal stem cells
ROS	Reactive oxygen species
hESCs	Human embryonic stem cells
