# Supplementary material for: Hypoxia Promotes the In Vitro Proliferation of Buffalo Spermatogonial Cells by Increasing Lactate and H3K18la Lactylation Levels
Source: Cells. 2025 Jun 3;14(11):832. doi: 10.3390/cells14110832 (PMC12154489; doi:10.3390/cells14110832)
Supplement: Supplementary file 1 [file cells-14-00832-s001.zip › cells-3659514-supplementary.pdf]

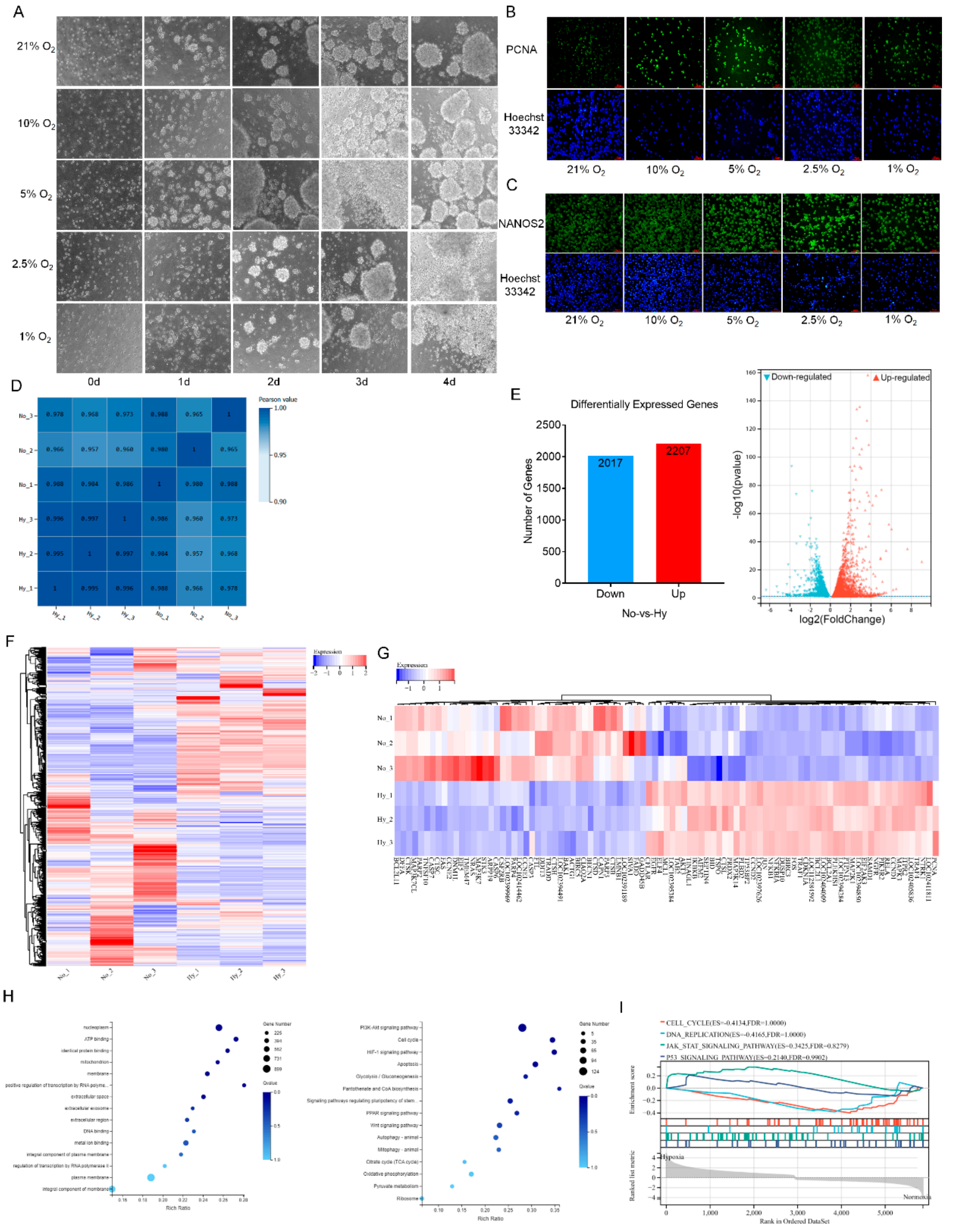

Figure S1: Effects of Oxygen Concentration on bSCs: Morphology, Protein Expression, Gene Correlation, Differential Expression, and Pathway Analysis. A: Pictures of bSCs cultured at different oxygen concentrations for different days taken under a bright field. B-C: After immunofluorescence staining, the expression and localization of the PCNA (B) and NANOS2 (C) proteins are displayed in different fluorescence color channels when the cells are cultured at different oxygen concentrations (green: target protein; blue: nuclear localization). D: To reflect the correlation of gene expression between samples, the Pearson correlation coefficient of

all gene expression levels between each pair of samples was calculated, and these coefficients are shown in the form of a heatmap. The correlation coefficient reflects the similarity of the overall gene expression between each sample. The higher the correlation coefficient is, the more similar the gene expression levels are. E: According to the gene expression levels of each sample, the detected significantly differentially expressed genes are plotted as histograms and volcano plots; F: FPKM of differentially expressed genes in each comparison group. The values were clustered, and a heatmap was drawn; G: Heatmap results of gene expression related to cell proliferation in the transcriptome sequencing results (No: normoxia, 21% oxygen concentration; Hy: hypoxia, 5% oxygen concentration); H: Through database comparison, the differentially expressed gene cluster analysis results were integrated, and a GO and KEGG pathway enrichment bubble chart was drawn. (Magnification: 200×, scale bar = 50 μm); I: GSEA results for the cell cycle, DNA replication, the JAK/STAT signaling pathway and the P53 signaling pathway. (Magnification: 200×, scale bar = 50 μm).

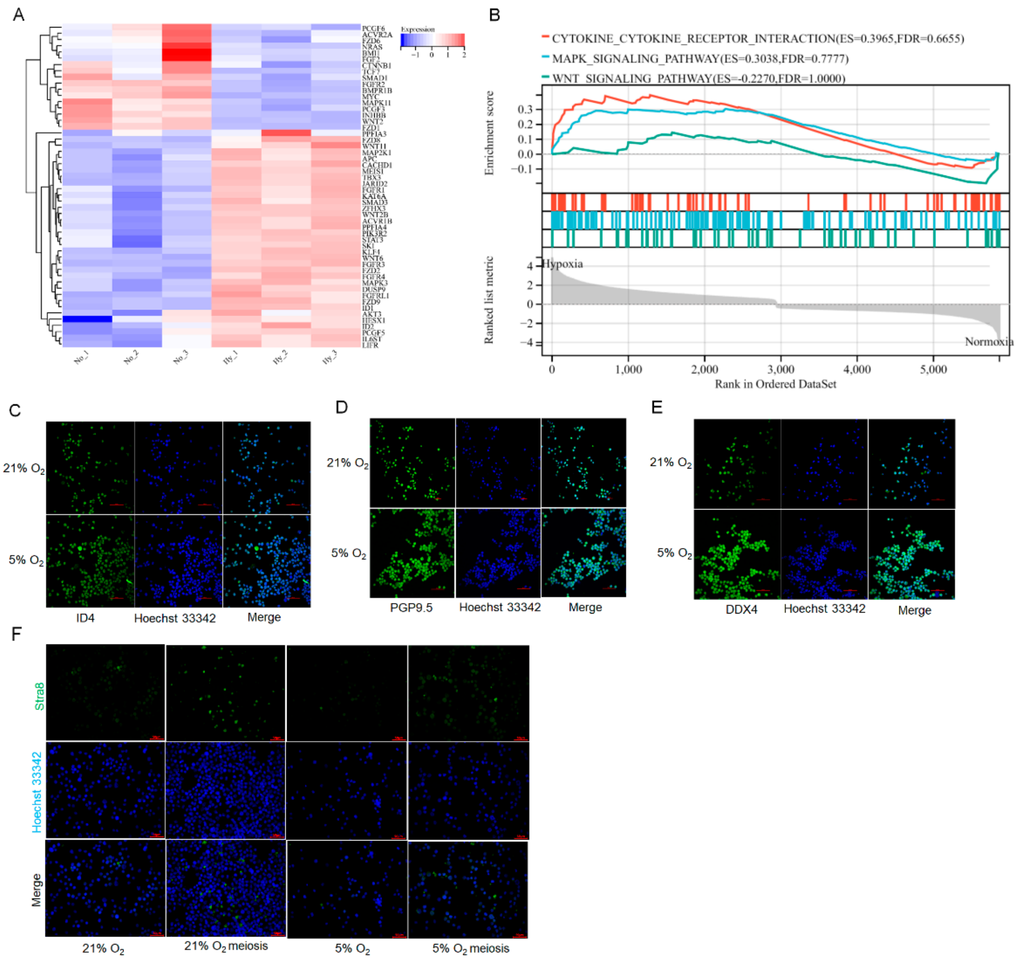

Figure S2: Transcriptomic and Immunofluorescence Analysis of Pluripotency and Meiosis-Related Proteins in bSCs under Normoxia and Hypoxia. A: Heatmap of gene expression related to stem cell pluripotency according to the transcriptomic sequencing results; B: GSEA results of cytokines, the MAPK signaling pathway and the WNT signaling pathway; C-E: Immunofluorescence staining was used to determine the expression and localization of the pluripotency protein ID4 (C) and the reproductive-specific proteins PGP9.5 (D) and DDX4 (E) in bSCs cultured under normoxia and hypoxia; F: Immunofluorescence staining was used to determine the expression of the meiosis-related protein Stra8 in bSCs cultured under normoxia and hypoxia before and after meiosis induction (green: target protein; blue: nuclear localization) (magnification: 200×; scale bar = 50 μm).

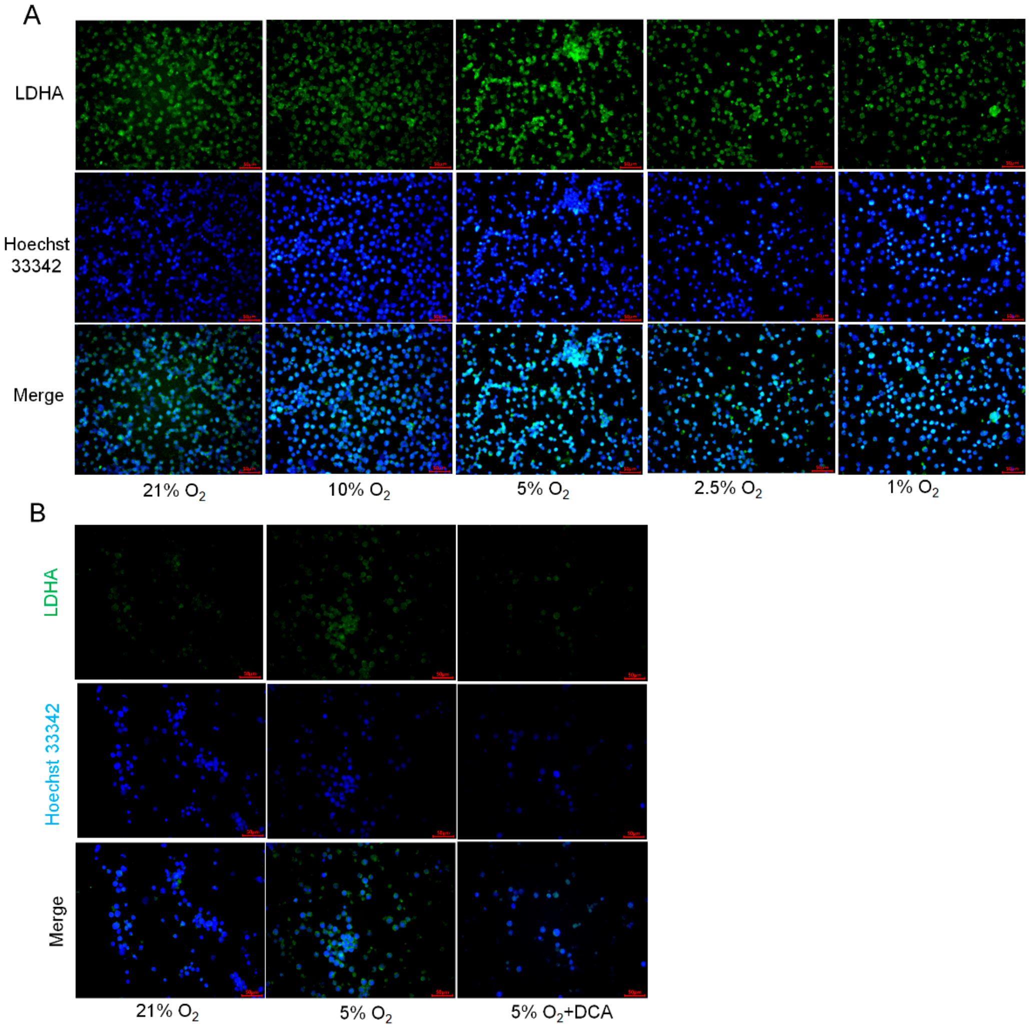

Figure S3: Multichannel immunofluorescence staining images. A: Expression and localization of LDHA, a key protein involved in glycolysis, in bSCs cultured with different oxygen concentrations. B: Expression and localization of the LDHA protein after normoxia, hypoxia, and hypoxia plus DCA blocked glycolysis (green: target protein; blue: nuclear localization) (magnification: 200×; scale bar = 50 μm).

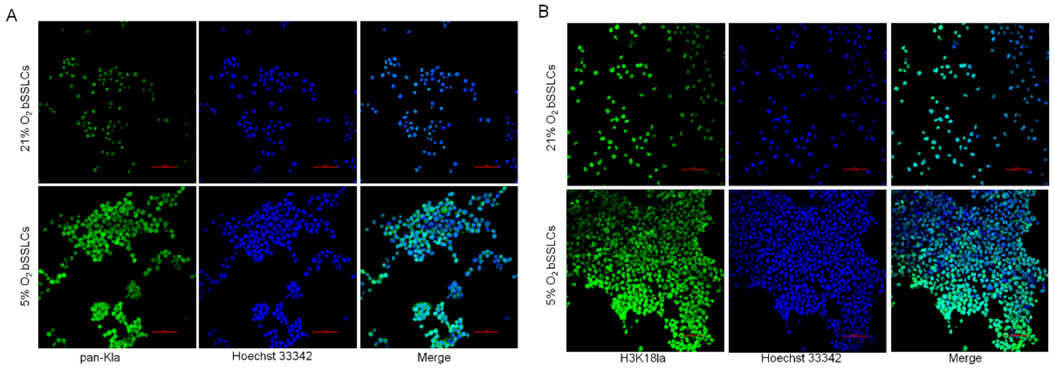

Figure S4: Multichannel Immunofluorescence of pan-Kla and H3K18la Protein Localization in bSCs under Normoxia and Hypoxia. Multichannel immunofluorescence images showing the localization of pan-Kla (A) and H3K18la (B) protein expression in bSCs cultured under normoxic and hypoxic conditions (green: target protein; blue: nuclear localization) (magnification: 200×; scale bar = 50 μm).

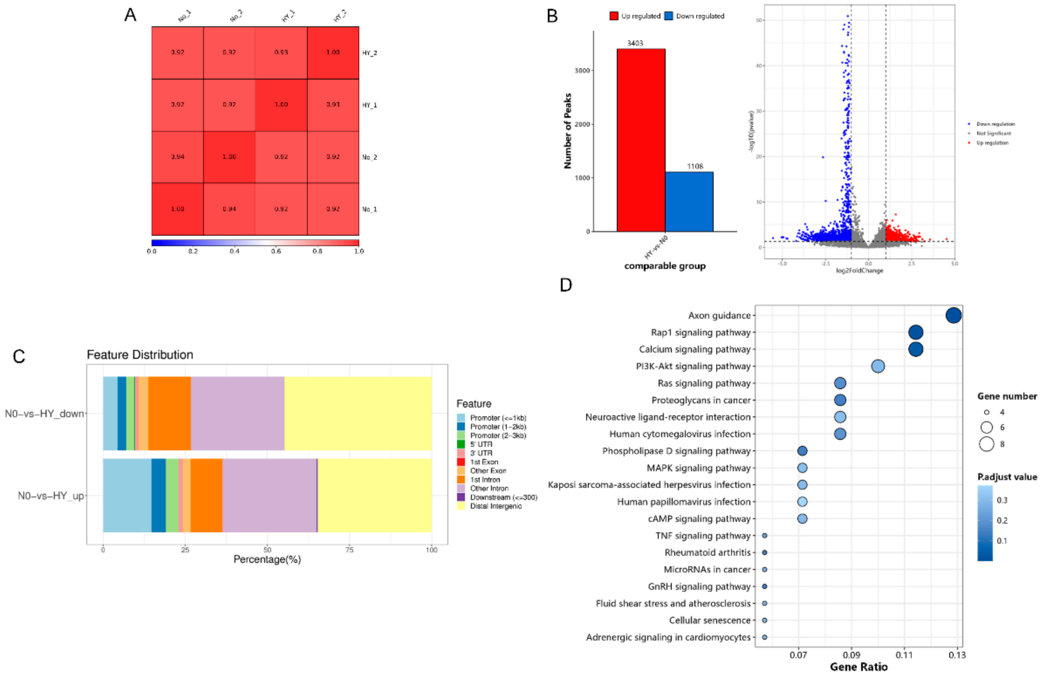

Figure S5: CUT&Tag and RNA-Seq Analysis: Correlation, Differential Peaks, Functional Annotation, and Pathway Enrichment. A: Heatmap of correlation between CUT&Tag sequencing samples; B: Statistical histogram and volcano plot of differential peak numbers in each comparison group; C: Histogram of functional region annotation distribution of differential peaks in all comparison groups; D: Top 20 significantly enriched differential peaks of neighboring genes and bubble chart of set GO and KEGG entries; E: Bubble chart of significantly enriched differentially expressed genes in GO and KEGG entries after CUT&Tag-seq combined with RNA-seq analysis.

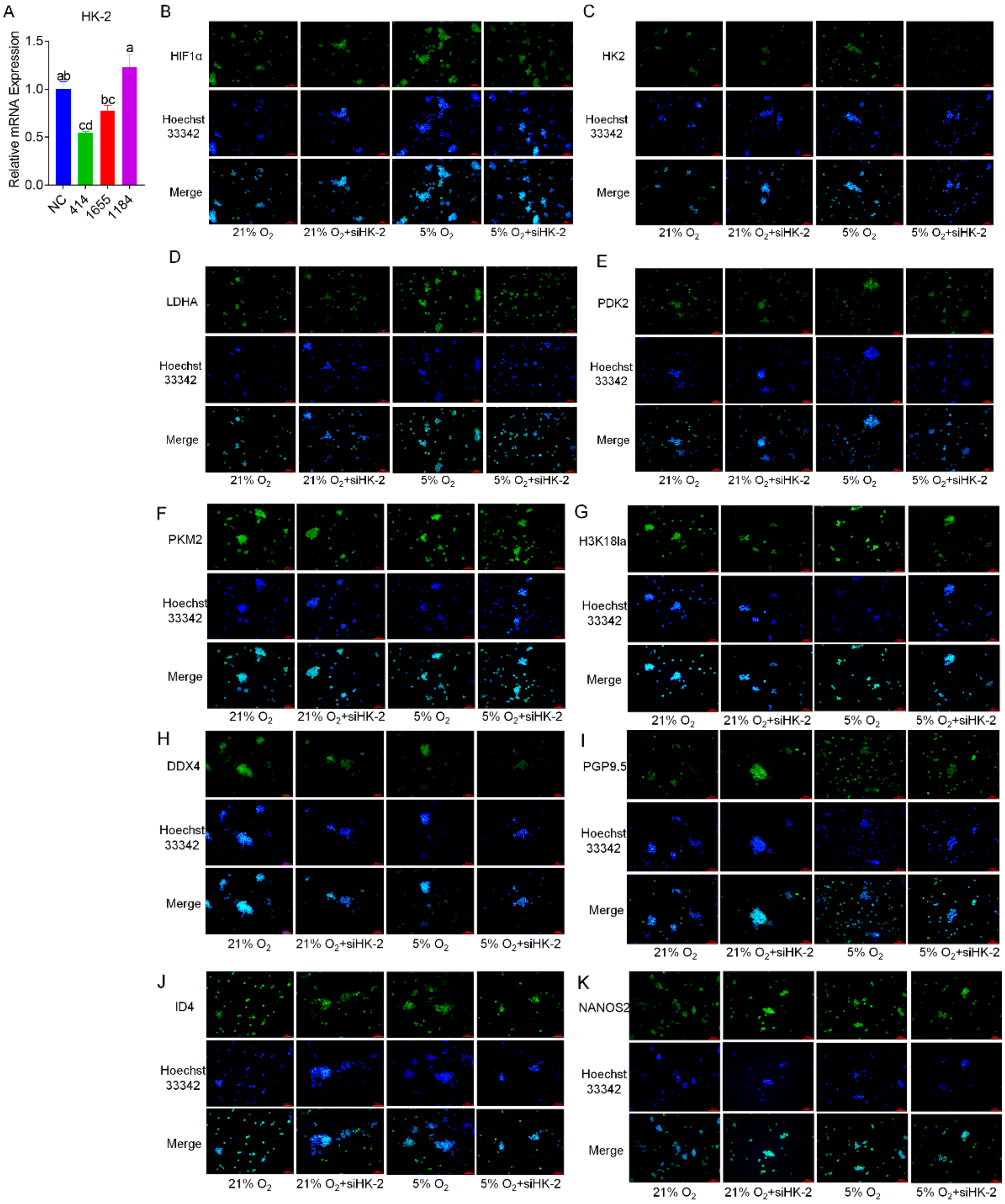

Figure S6: Effects of HK-2 Knockdown on Glycolytic Proteins, Histone Lactylation, and Stem Cell Markers in bSCs under Normoxia and Hypoxia. A: qPCR analysis of the effect of siRNA transfection of three different HK-2 interference sites on HK-2 expression; B-K: Multichannel immunofluorescence staining images showing the expression and localization of each functional protein after HK-2 expression was disrupted under normoxic and hypoxic conditions. Key proteins of the glycolytic pathway: HIF1α (B), HK2 (C), LDHA (D), PDK2 (E), PKM2 (F); histone lactylation modification site H3K18la (G); cell reproduction-specific proteins DDX4 (H) and PGP9.5 (I); pluripotency protein ID4 (J); SSC-specific protein NANOS2 (K). [Green: target protein; blue: nuclear localization) (magnification: 200×; scale bar = 50 μm; different letters represent significant differences between groups ($P < 0.05$)].
